# 1,1′-(*p*-Phenyl­enedimethyl­idene)diimidazol-3-ium bis­{2-[(2-carb­oxy­phen­yl)disulfan­yl]benzoate} dihydrate

**DOI:** 10.1107/S1600536810047021

**Published:** 2010-11-20

**Authors:** Zhengming Liu, Qiang Liu, Limin Yuan, Wenlong Liu

**Affiliations:** a Testing Center, Yangzhou University, Yangzhou 225002, People’s Republic of China; b College of Chemistry and Chemical Engineering, Yangzhou University, Yangzhou 225002, People’s Republic of China

## Abstract

The title salt, C_14_H_16_N_4_^2+^·2C_14_H_9_O_4_S_2_^−^·2H_2_O, was obtained by the co-crystalization of 2,2′-dithio­dibenzoic acid with 1,4-bis­(imidazol-1-ylmeth­yl)benzene. It consists of 2-[(2-carb­oxy­phen­yl)disulfan­yl]benzoate anions, centrosymmetric 1,1′-(*p*-phenyl­enedimethyl­idene)diimidazol-3-ium cations and water mol­ecules. O—H⋯O, O—H⋯S and N—H⋯O hydrogen-bonding inter­actions among the components lead to the formation of a three-dimensional network.

## Related literature

For background to the co-crystalization of 2,2′-dithio­dibenzoic acid with bipyridine-type mol­ecules, see: Bi *et al.* (2002 ▶); Broker & Tiekink (2007 ▶); Broker *et al.* (2008 ▶); Hu *et al.* (2004 ▶).
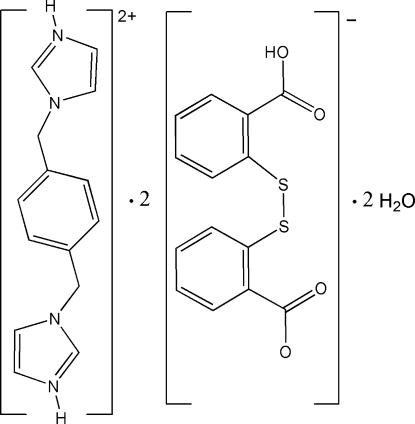


## Experimental

### 

#### Crystal data

C_14_H_16_N_4_^2+^·2C_14_H_9_O_4_S_2_^−^·2H_2_O
*M* *_r_* = 887.00
Triclinic, 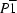

*a* = 4.6776 (11) Å
*b* = 12.201 (3) Å
*c* = 18.850 (4) Å
α = 107.985 (3)°
β = 90.686 (3)°
γ = 100.634 (3)°
*V* = 1002.9 (4) Å^3^
*Z* = 1
Mo *K*α radiation
μ = 0.30 mm^−1^
*T* = 296 K
0.45 × 0.43 × 0.38 mm

#### Data collection

Bruker SMART APEX CCD diffractometer
Absorption correction: multi-scan (*SADABS*; Sheldrick, 2004 ▶) *T*_min_ = 0.876, *T*_max_ = 0.894
7640 measured reflections
3708 independent reflections
2720 reflections with *I* > 2σ(*I*)
*R*_int_ = 0.030

#### Refinement

*R*[*F* ^2^ > 2σ(*F* ^2^)] = 0.044
*wR*(*F* ^2^) = 0.134
*S* = 1.08
3708 reflections
284 parameters
9 restraints
H atoms treated by a mixture of independent and constrained refinement
Δρ_max_ = 0.26 e Å^−3^
Δρ_min_ = −0.24 e Å^−3^

### 

Data collection: *SMART* (Bruker, 2002 ▶); cell refinement: *SAINT-Plus* (Bruker, 2003 ▶); data reduction: *SAINT-Plus*; program(s) used to solve structure: *SHELXS97* (Sheldrick, 2008 ▶); program(s) used to refine structure: *SHELXL97* (Sheldrick, 2008 ▶); molecular graphics: *SHELXTL* (Sheldrick, 2008 ▶) and *DIAMOND* (Brandenburg, 2006 ▶); software used to prepare material for publication: *publCIF* (Westrip, 2010 ▶).

## Supplementary Material

Crystal structure: contains datablocks I, global. DOI: 10.1107/S1600536810047021/ng5063sup1.cif

Structure factors: contains datablocks I. DOI: 10.1107/S1600536810047021/ng5063Isup2.hkl

Additional supplementary materials:  crystallographic information; 3D view; checkCIF report

## Figures and Tables

**Table 1:** Hydrogen-bond geometry (Å, °)

*D*—H⋯*A*	*D*—H	H⋯*A*	*D*⋯*A*	*D*—H⋯*A*
O1*W*—H1*WB*⋯S1^i^	0.88 (2)	2.80 (3)	3.507 (3)	138 (4)
O1*W*—H1*WB*⋯O2^i^	0.88 (2)	2.30 (2)	3.141 (4)	159 (4)
N1—H1⋯O1^ii^	0.91 (2)	1.75 (2)	2.657 (3)	176 (3)
O4—H4*A*⋯O1^ii^	0.87 (2)	1.73 (3)	2.567 (3)	161 (4)
O1*W*—H1*WA*⋯O2	0.88 (2)	1.94 (2)	2.811 (3)	171 (4)

Symmetry codes: (i) 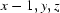; (ii) 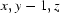.

**Fig. 1.**
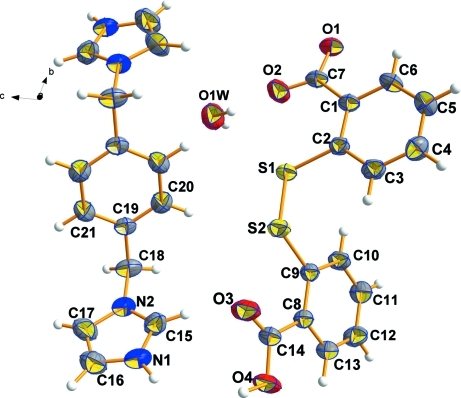
Molecular structures of the title compound, showing atom-labelling scheme and displacement ellipsoids at the 50% probability level.

**Fig. 2.**
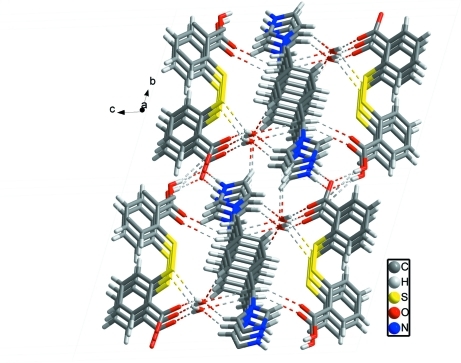
The three-dimensional packing structure of the title compound, Hydrogen bonds are shown as dashed lines.

## supplementary crystallographic information

### Comment

The dicarboxylic acid DTBA has been shown to be effective and reliable in forming a range of co-crystals with a series of bipyridine-type molecules leading to varying supramolecular architectures(Broker & Tiekink, 2007; Broker *et al.*, 2008). In comparison, the use of biimidazole-type molecules to co-crystal with DTBA remains largely unexplored. Herein, the formation of co-crystals of 2,2'-dithiodibenzoic acid with 1,4-bis(imidazol-1-ylmethyl)benzene is described, which were isolated from methanol. The asymmetric unit of the title compound comprises a singly deprotonated DTBA anion, half a 1,4-bis(imidazolium-1-ylmethyl)benzene dication, disposed about a centre of inversion, and a solvent water molecule of crystallization (Fig. 1). The dihedral angle between the two phenyl rings of HDTBA^- ^and torsion angle (C2/S1/S2/C9) are 73.54 (7)° and -84.93 (12)°, respectively. There are extensive hydrogen-bonding interactions between the carboxyl groups, protonated N atoms and water molecules of (I). As shown in Fig. 2, hydrogen-bonding interactions link water molecules to (C_14_H_9_O_4_S_2_)^-^ anions, (C_14_H_16_N_4_)^2+^ cations and connect (C_14_H_9_O_4_S_2_)^- ^anions to (C_14_H_16_N_4_)^2+^ cations to form an extended three-dimensional network.

### Experimental

2,2'-Dithiodibenzoic acid (153 mg, 0.5 mmol) was dissolved in 15 ml me thanol, and a solution of 1,4-bis(imidazol-1-ylmethyl)benzene (191 mg, 0.8 mmol) in 20 ml me thanol was added dropwise under intense agitation. The resulting mixture was stirred under reflux conditions for 1 h and allowed to cool to room temperature, filtered. After allowing the solution to stand for five days, colourless block-like crystals of (I) were obtained in 42% yield.

### Refinement

The O-bound and N-bound H atoms were located in difference Fourier maps and were refined with the distance restraints O—H = 0.84±0.02 Å, N—H = 0.87±0.03 Å, and with *U*_iso_(H) = 1.5*U*_eq_(O). The temperature factor of N-bound H atom was refined.

Carbon-bound H-atoms were placed in calculated positions (C—H 0.93–0.97 Å) and were included in the refinement in the riding model approximation, with *U*_iso_(H) set to 1.2*U*_eq_(C).

The refinement of O-bound and N-bound H atoms and the C—C distances in the phenylene ring were performed using 9 least-squares restraints by applying *DFIX* instructions of *SHELXTL*.

### Crystal data

**Table:**

C_14_H_16_N_4_^2+^·2C_14_H_9_O_4_S_2_^−^·2H_2_O	*Z* = 1
*M**_r_* = 887.00	*F*(000) = 462
Triclinic, *P*1	*D*_x_ = 1.469 Mg m^−^^3^
Hall symbol: -P 1	Mo *K*α radiation, λ = 0.71073 Å
*a* = 4.6776 (11) Å	Cell parameters from 1975 reflections
*b* = 12.201 (3) Å	θ = 2.3–24.5°
*c* = 18.850 (4) Å	µ = 0.30 mm^−^^1^
α = 107.985 (3)°	*T* = 296 K
β = 90.686 (3)°	Block, colourless
γ = 100.634 (3)°	0.45 × 0.43 × 0.38 mm
*V* = 1002.9 (4) Å^3^	

### Data collection

**Table:**

Bruker SMART APEX CCD diffractometer	3708 independent reflections
Radiation source: fine-focus sealed tube	2720 reflections with *I* > 2σ(*I*)
graphite	*R*_int_ = 0.030
φ and ω scans	θ_max_ = 25.5°, θ_min_ = 1.8°
Absorption correction: multi-scan (*SADABS*; Sheldrick, 2004)	*h* = −5→5
*T*_min_ = 0.876, *T*_max_ = 0.894	*k* = −14→14
7640 measured reflections	*l* = −20→22

### Refinement

**Table:**

Refinement on *F*^2^	Primary atom site location: structure-invariant direct methods
Least-squares matrix: full	Secondary atom site location: difference Fourier map
*R*[*F*^2^ > 2σ(*F*^2^)] = 0.044	Hydrogen site location: inferred from neighbouring sites
*wR*(*F*^2^) = 0.134	H atoms treated by a mixture of independent and constrained refinement
*S* = 1.08	*w* = 1/[σ^2^(*F*_o_^2^) + (0.0676*P*)^2^ + 0.1163*P*] where *P* = (*F*_o_^2^ + 2*F*_c_^2^)/3
3708 reflections	(Δ/σ)_max_ < 0.001
284 parameters	Δρ_max_ = 0.26 e Å^−^^3^
9 restraints	Δρ_min_ = −0.24 e Å^−^^3^

### Special details

**Table:**

Geometry. All e.s.d.'s (except the e.s.d. in the dihedral angle between two l.s. planes) are estimated using the full covariance matrix. The cell e.s.d.'s are taken into account individually in the estimation of e.s.d.'s in distances, angles and torsion angles; correlations between e.s.d.'s in cell parameters are only used when they are defined by crystal symmetry. An approximate (isotropic) treatment of cell e.s.d.'s is used for estimating e.s.d.'s involving l.s. planes.
Refinement. Refinement of *F*^2^ against ALL reflections. The weighted *R*-factor *wR* and goodness of fit *S* are based on *F*^2^, conventional *R*-factors *R* are based on *F*, with *F* set to zero for negative *F*^2^. The threshold expression of *F*^2^ > σ(*F*^2^) is used only for calculating *R*-factors(gt) *etc*. and is not relevant to the choice of reflections for refinement. *R*-factors based on *F*^2^ are statistically about twice as large as those based on *F*, and *R*- factors based on ALL data will be even larger.

### Fractional atomic coordinates and isotropic or equivalent isotropic displacement parameters (Å^2^)

**Table:**

	*x*	*y*	*z*	*U*_iso_*/*U*_eq_	
C1	0.9224 (5)	0.7640 (2)	0.21093 (14)	0.0356 (6)	
C2	0.9027 (5)	0.6438 (2)	0.19945 (14)	0.0352 (6)	
C3	1.0412 (6)	0.5796 (2)	0.14067 (15)	0.0443 (7)	
H3	1.0227	0.4990	0.1313	0.053*	
C4	1.2053 (6)	0.6331 (2)	0.09606 (16)	0.0500 (7)	
H4	1.2968	0.5885	0.0571	0.060*	
C5	1.2351 (6)	0.7522 (3)	0.10862 (17)	0.0483 (7)	
H5	1.3507	0.7889	0.0794	0.058*	
C6	1.0910 (6)	0.8157 (2)	0.16511 (16)	0.0437 (7)	
H6	1.1066	0.8958	0.1729	0.052*	
C7	0.7699 (6)	0.8391 (2)	0.27118 (16)	0.0405 (6)	
C8	0.5723 (5)	0.1938 (2)	0.13953 (14)	0.0375 (6)	
C9	0.5950 (5)	0.3156 (2)	0.15799 (14)	0.0357 (6)	
C10	0.4493 (6)	0.3591 (2)	0.11132 (15)	0.0433 (7)	
H10	0.4668	0.4399	0.1224	0.052*	
C11	0.2789 (6)	0.2841 (3)	0.04877 (17)	0.0496 (7)	
H11	0.1817	0.3148	0.0184	0.060*	
C12	0.2513 (6)	0.1640 (3)	0.03089 (17)	0.0530 (8)	
H12	0.1350	0.1135	−0.0110	0.064*	
C13	0.3991 (6)	0.1202 (2)	0.07623 (16)	0.0461 (7)	
H13	0.3825	0.0393	0.0642	0.055*	
C14	0.7222 (6)	0.1414 (2)	0.18676 (16)	0.0445 (7)	
S1	0.69255 (16)	0.57314 (5)	0.25775 (4)	0.0440 (2)	
S2	0.81448 (17)	0.41403 (6)	0.23901 (4)	0.0461 (2)	
O1	0.7530 (4)	0.94073 (15)	0.26811 (11)	0.0504 (5)	
O2	0.6751 (5)	0.80190 (17)	0.32150 (12)	0.0636 (6)	
O3	0.8747 (5)	0.19838 (17)	0.24241 (13)	0.0676 (7)	
O4	0.6690 (6)	0.02564 (17)	0.16259 (13)	0.0670 (7)	
H4A	0.737 (8)	0.003 (3)	0.1976 (19)	0.100*	
C15	0.3094 (6)	0.1496 (3)	0.35488 (17)	0.0514 (7)	
H15	0.3404	0.1817	0.3162	0.062*	
C16	0.3162 (9)	0.0374 (3)	0.4231 (2)	0.0735 (10)	
H16	0.3513	−0.0234	0.4398	0.088*	
C17	0.1658 (8)	0.1195 (3)	0.4573 (2)	0.0731 (11)	
H17	0.0816	0.1277	0.5026	0.088*	
C18	0.0401 (6)	0.2974 (2)	0.43199 (18)	0.0513 (7)	
H18A	−0.1118	0.2936	0.4662	0.062*	
H18B	−0.0463	0.3040	0.3868	0.062*	
C19	0.2748 (5)	0.4039 (2)	0.46723 (14)	0.0406 (6)	
C20	0.4029 (7)	0.4741 (3)	0.42709 (17)	0.0623 (9)	
H20	0.3376	0.4576	0.3774	0.075*	
C21	0.3731 (7)	0.4311 (3)	0.54080 (16)	0.0656 (9)	
H21	0.2879	0.3853	0.5692	0.079*	
N1	0.4076 (5)	0.0579 (2)	0.36030 (15)	0.0512 (6)	
H1	0.519 (5)	0.016 (2)	0.3270 (14)	0.056 (9)*	
N2	0.1594 (5)	0.18873 (19)	0.41342 (13)	0.0466 (6)	
O1W	0.2006 (6)	0.7532 (2)	0.40459 (14)	0.0758 (7)	
H1WA	0.351 (5)	0.761 (4)	0.378 (2)	0.114*	
H1WB	0.049 (5)	0.747 (4)	0.374 (2)	0.114*	

### Atomic displacement parameters (Å^2^)

**Table:**

	*U*^11^	*U*^22^	*U*^33^	*U*^12^	*U*^13^	*U*^23^
C1	0.0361 (14)	0.0302 (13)	0.0407 (15)	0.0070 (11)	−0.0009 (11)	0.0114 (11)
C2	0.0401 (14)	0.0307 (13)	0.0356 (14)	0.0101 (11)	0.0003 (11)	0.0098 (11)
C3	0.0482 (16)	0.0360 (14)	0.0505 (17)	0.0155 (12)	0.0083 (13)	0.0120 (13)
C4	0.0498 (17)	0.0509 (17)	0.0504 (18)	0.0191 (14)	0.0140 (14)	0.0121 (14)
C5	0.0459 (16)	0.0509 (17)	0.0537 (18)	0.0078 (13)	0.0100 (14)	0.0252 (14)
C6	0.0470 (16)	0.0321 (14)	0.0559 (18)	0.0072 (12)	0.0038 (14)	0.0201 (13)
C7	0.0451 (16)	0.0284 (13)	0.0477 (16)	0.0093 (11)	0.0017 (13)	0.0105 (12)
C8	0.0435 (15)	0.0298 (13)	0.0393 (15)	0.0086 (11)	0.0084 (12)	0.0103 (12)
C9	0.0403 (14)	0.0293 (13)	0.0380 (14)	0.0082 (11)	0.0092 (11)	0.0103 (11)
C10	0.0503 (16)	0.0351 (14)	0.0475 (16)	0.0142 (12)	0.0005 (13)	0.0141 (13)
C11	0.0501 (17)	0.0482 (17)	0.0500 (18)	0.0097 (14)	−0.0029 (14)	0.0148 (14)
C12	0.0528 (18)	0.0459 (17)	0.0484 (18)	−0.0006 (14)	−0.0066 (14)	0.0043 (14)
C13	0.0561 (17)	0.0312 (14)	0.0462 (17)	0.0035 (13)	0.0066 (14)	0.0083 (13)
C14	0.0612 (18)	0.0312 (14)	0.0445 (17)	0.0142 (13)	0.0093 (14)	0.0137 (13)
S1	0.0631 (5)	0.0266 (3)	0.0436 (4)	0.0107 (3)	0.0119 (3)	0.0114 (3)
S2	0.0665 (5)	0.0276 (3)	0.0438 (4)	0.0100 (3)	−0.0065 (3)	0.0108 (3)
O1	0.0689 (13)	0.0263 (9)	0.0578 (12)	0.0160 (9)	0.0077 (10)	0.0122 (9)
O2	0.0914 (16)	0.0426 (12)	0.0683 (15)	0.0263 (11)	0.0380 (13)	0.0250 (11)
O3	0.1023 (18)	0.0354 (11)	0.0633 (15)	0.0134 (11)	−0.0240 (13)	0.0143 (11)
O4	0.1058 (19)	0.0289 (10)	0.0678 (15)	0.0140 (11)	−0.0092 (13)	0.0178 (10)
C15	0.0584 (19)	0.0508 (18)	0.0452 (17)	0.0190 (15)	0.0045 (15)	0.0108 (14)
C16	0.104 (3)	0.0493 (19)	0.079 (3)	0.0279 (19)	0.024 (2)	0.0288 (19)
C17	0.106 (3)	0.0486 (19)	0.074 (2)	0.0176 (19)	0.040 (2)	0.0295 (18)
C18	0.0451 (16)	0.0388 (15)	0.0627 (19)	0.0098 (13)	0.0029 (14)	0.0049 (14)
C19	0.0419 (15)	0.0333 (14)	0.0432 (16)	0.0086 (11)	0.0046 (12)	0.0067 (12)
C20	0.076 (2)	0.057 (2)	0.0456 (18)	−0.0079 (17)	−0.0084 (16)	0.0171 (16)
C21	0.085 (2)	0.0540 (19)	0.0502 (19)	−0.0174 (17)	−0.0008 (17)	0.0245 (16)
N1	0.0537 (15)	0.0364 (13)	0.0564 (16)	0.0104 (11)	0.0033 (13)	0.0034 (12)
N2	0.0457 (13)	0.0360 (13)	0.0523 (15)	0.0053 (10)	0.0051 (11)	0.0070 (11)
O1W	0.0771 (17)	0.0846 (18)	0.0666 (17)	0.0079 (15)	0.0150 (13)	0.0294 (14)

### Geometric parameters (Å, °)

**Table:**

C1—C6	1.391 (3)	C14—O3	1.200 (3)
C1—C2	1.400 (3)	C14—O4	1.317 (3)
C1—C7	1.502 (4)	S1—S2	2.0515 (10)
C2—C3	1.389 (3)	O4—H4A	0.87 (2)
C2—S1	1.793 (3)	C15—N1	1.316 (4)
C3—C4	1.376 (4)	C15—N2	1.325 (4)
C3—H3	0.9300	C15—H15	0.9300
C4—C5	1.378 (4)	C16—C17	1.337 (5)
C4—H4	0.9300	C16—N1	1.342 (4)
C5—C6	1.375 (4)	C16—H16	0.9300
C5—H5	0.9300	C17—N2	1.356 (4)
C6—H6	0.9300	C17—H17	0.9300
C7—O2	1.226 (3)	C18—N2	1.478 (3)
C7—O1	1.276 (3)	C18—C19	1.503 (4)
C8—C13	1.391 (4)	C18—H18A	0.9700
C8—C9	1.400 (3)	C18—H18B	0.9700
C8—C14	1.482 (4)	C19—C20	1.374 (3)
C9—C10	1.389 (4)	C19—C21	1.374 (3)
C9—S2	1.790 (3)	C20—C21^i^	1.381 (3)
C10—C11	1.380 (4)	C20—H20	0.9300
C10—H10	0.9300	C21—C20^i^	1.381 (3)
C11—C12	1.379 (4)	C21—H21	0.9300
C11—H11	0.9300	N1—H1	0.911 (17)
C12—C13	1.378 (4)	O1W—H1WA	0.876 (18)
C12—H12	0.9300	O1W—H1WB	0.883 (18)
C13—H13	0.9300		
			
C6—C1—C2	118.7 (2)	C8—C13—H13	119.2
C6—C1—C7	118.9 (2)	O3—C14—O4	122.8 (3)
C2—C1—C7	122.4 (2)	O3—C14—C8	123.6 (2)
C3—C2—C1	118.8 (2)	O4—C14—C8	113.6 (3)
C3—C2—S1	120.61 (19)	C2—S1—S2	106.04 (8)
C1—C2—S1	120.52 (19)	C9—S2—S1	105.87 (8)
C4—C3—C2	121.1 (2)	C14—O4—H4A	107 (3)
C4—C3—H3	119.5	N1—C15—N2	109.0 (3)
C2—C3—H3	119.5	N1—C15—H15	125.5
C3—C4—C5	120.5 (3)	N2—C15—H15	125.5
C3—C4—H4	119.7	C17—C16—N1	108.1 (3)
C5—C4—H4	119.7	C17—C16—H16	125.9
C6—C5—C4	118.8 (3)	N1—C16—H16	125.9
C6—C5—H5	120.6	C16—C17—N2	107.0 (3)
C4—C5—H5	120.6	C16—C17—H17	126.5
C5—C6—C1	122.0 (2)	N2—C17—H17	126.5
C5—C6—H6	119.0	N2—C18—C19	111.0 (2)
C1—C6—H6	119.0	N2—C18—H18A	109.4
O2—C7—O1	123.3 (2)	C19—C18—H18A	109.4
O2—C7—C1	119.2 (2)	N2—C18—H18B	109.4
O1—C7—C1	117.5 (2)	C19—C18—H18B	109.4
C13—C8—C9	119.1 (2)	H18A—C18—H18B	108.0
C13—C8—C14	119.2 (2)	C20—C19—C21	118.3 (2)
C9—C8—C14	121.7 (2)	C20—C19—C18	121.7 (2)
C10—C9—C8	118.8 (2)	C21—C19—C18	120.0 (2)
C10—C9—S2	120.34 (19)	C19—C20—C21^i^	121.2 (3)
C8—C9—S2	120.83 (19)	C19—C20—H20	119.4
C11—C10—C9	120.9 (3)	C21^i^—C20—H20	119.4
C11—C10—H10	119.6	C19—C21—C20^i^	120.5 (3)
C9—C10—H10	119.6	C19—C21—H21	119.7
C12—C11—C10	120.7 (3)	C20^i^—C21—H21	119.7
C12—C11—H11	119.7	C15—N1—C16	108.1 (3)
C10—C11—H11	119.7	C15—N1—H1	125.3 (19)
C13—C12—C11	118.8 (3)	C16—N1—H1	126.6 (19)
C13—C12—H12	120.6	C15—N2—C17	107.8 (3)
C11—C12—H12	120.6	C15—N2—C18	125.6 (3)
C12—C13—C8	121.7 (3)	C17—N2—C18	126.2 (3)
C12—C13—H13	119.2	H1WA—O1W—H1WB	104 (3)
			
C6—C1—C2—C3	−2.9 (4)	C14—C8—C13—C12	179.1 (3)
C7—C1—C2—C3	177.6 (2)	C13—C8—C14—O3	−179.7 (3)
C6—C1—C2—S1	179.13 (19)	C9—C8—C14—O3	−1.2 (4)
C7—C1—C2—S1	−0.4 (3)	C13—C8—C14—O4	−0.7 (4)
C1—C2—C3—C4	2.6 (4)	C9—C8—C14—O4	177.8 (2)
S1—C2—C3—C4	−179.4 (2)	C3—C2—S1—S2	16.6 (2)
C2—C3—C4—C5	−0.2 (4)	C1—C2—S1—S2	−165.39 (19)
C3—C4—C5—C6	−1.9 (4)	C10—C9—S2—S1	16.4 (2)
C4—C5—C6—C1	1.6 (4)	C8—C9—S2—S1	−165.10 (18)
C2—C1—C6—C5	0.8 (4)	C2—S1—S2—C9	−84.96 (12)
C7—C1—C6—C5	−179.6 (3)	N1—C16—C17—N2	1.7 (4)
C6—C1—C7—O2	−164.2 (3)	N2—C18—C19—C20	100.9 (3)
C2—C1—C7—O2	15.3 (4)	N2—C18—C19—C21	−77.0 (3)
C6—C1—C7—O1	13.9 (4)	C21—C19—C20—C21^i^	0.8 (5)
C2—C1—C7—O1	−166.5 (2)	C18—C19—C20—C21^i^	−177.2 (3)
C13—C8—C9—C10	−1.6 (4)	C20—C19—C21—C20^i^	−0.8 (5)
C14—C8—C9—C10	179.9 (2)	C18—C19—C21—C20^i^	177.2 (3)
C13—C8—C9—S2	179.85 (19)	N2—C15—N1—C16	0.6 (4)
C14—C8—C9—S2	1.4 (3)	C17—C16—N1—C15	−1.5 (4)
C8—C9—C10—C11	1.6 (4)	N1—C15—N2—C17	0.5 (4)
S2—C9—C10—C11	−179.8 (2)	N1—C15—N2—C18	173.2 (2)
C9—C10—C11—C12	−0.5 (4)	C16—C17—N2—C15	−1.4 (4)
C10—C11—C12—C13	−0.6 (4)	C16—C17—N2—C18	−174.1 (3)
C11—C12—C13—C8	0.6 (4)	C19—C18—N2—C15	−78.1 (3)
C9—C8—C13—C12	0.5 (4)	C19—C18—N2—C17	93.3 (4)

Symmetry codes: (i) −*x*+1, −*y*+1, −*z*+1.

### Hydrogen-bond geometry (Å, °)

**Table:**

*D*—H···*A*	*D*—H	H···*A*	*D*···*A*	*D*—H···*A*
O1W—H1WB···S1^ii^	0.88 (2)	2.80 (3)	3.507 (3)	138 (4)
O1W—H1WB···O2^ii^	0.88 (2)	2.30 (2)	3.141 (4)	159 (4)
N1—H1···O1^iii^	0.91 (2)	1.75 (2)	2.657 (3)	176 (3)
O4—H4A···O1^iii^	0.87 (2)	1.73 (3)	2.567 (3)	161 (4)
O1W—H1WA···O2	0.88 (2)	1.94 (2)	2.811 (3)	171 (4)

Symmetry codes: (ii) *x*−1, *y*, *z*; (iii) *x*, *y*−1, *z*.
